# Cemental tear on maxillary first molars related to traumatic occlusion: a case report

**DOI:** 10.3389/froh.2025.1686579

**Published:** 2025-12-04

**Authors:** Di Wu, Xueyu Liu, Degang Sun, Dashan Wang, Lingxiang Wang

**Affiliations:** 1 Qingdao Stomatological Hospital Affiliated to Qingdao University, Qingdao, China; 2 The Affiliated Hospital of Qingdao University, Qingdao, China; 3 School of Stomatology, Qingdao University, Qingdao, China

**Keywords:** cemental tear, maxillary first molars, cone-beam computed tomography (CBCT), traumatic occlusion, oro-facial pain

## Abstract

A cemental tear represents a unique type of root surface fracture associated with the destruction of periodontal and periapical tissues. This report presents a case of cemental tears in maxillary first molars. Based on clinical and radiographic evidence, a tentative diagnosis of chronic periapical periodontitis (cemental tear) was made for both the left and right maxillary first molars. After careful consideration, the patient postponed treatment of the asymptomatic right maxillary first molar. The left maxillary first molar due to severe alveolar bone loss, was extracted seven days later. Early diagnosis and complete removal of the cemental tear are key factors in successful treatment. Therefore, cemental tears should be considered in all teeth, including molars. Clinicians should remain vigilant when examining patients presenting with potential causative factors such as localized deep periodontal pockets, loss of attachment, or signs of occlusal trauma.

## 1 Introduction

A cemental tear is a special kind of root surface fracture resulting in partial or complete separation of cementum from the root surface (1), which may further lead to the breakdown of periodontal and periapical tissues. It is challenging for clinicians to diagnose cemental tears on time, resulting in delayed treatment and compromising the prognosis. Cemental tears are reported more frequently in incisors or premolars than in molars (2). Possible predisposing factors include gender, age, tooth type, and traumatic occlusal forces. The morphology of cemental tears can be either piece-shaped or other shapes (2). Clinically, cemental tear shows a deep, narrow periodontal pocket mimicking localized periodontitis, apical periodontitis, or vertical root fractures (3). Partial detachment of the cementum cannot be detected solely by conventional radiography. However, a cemental tear detached from the root surface can be diagnosed by conventional and cone-beam computed tomography. Therefore, a comprehensive clinical and radiological examination and a thorough history are essential for early diagnosis and treatment planning. We have presented a rare case of bilateral cemental tears on the right and left maxillary first molars and its management with periodontal and surgical interventions.

## 2 Case report

**Figure 1:**
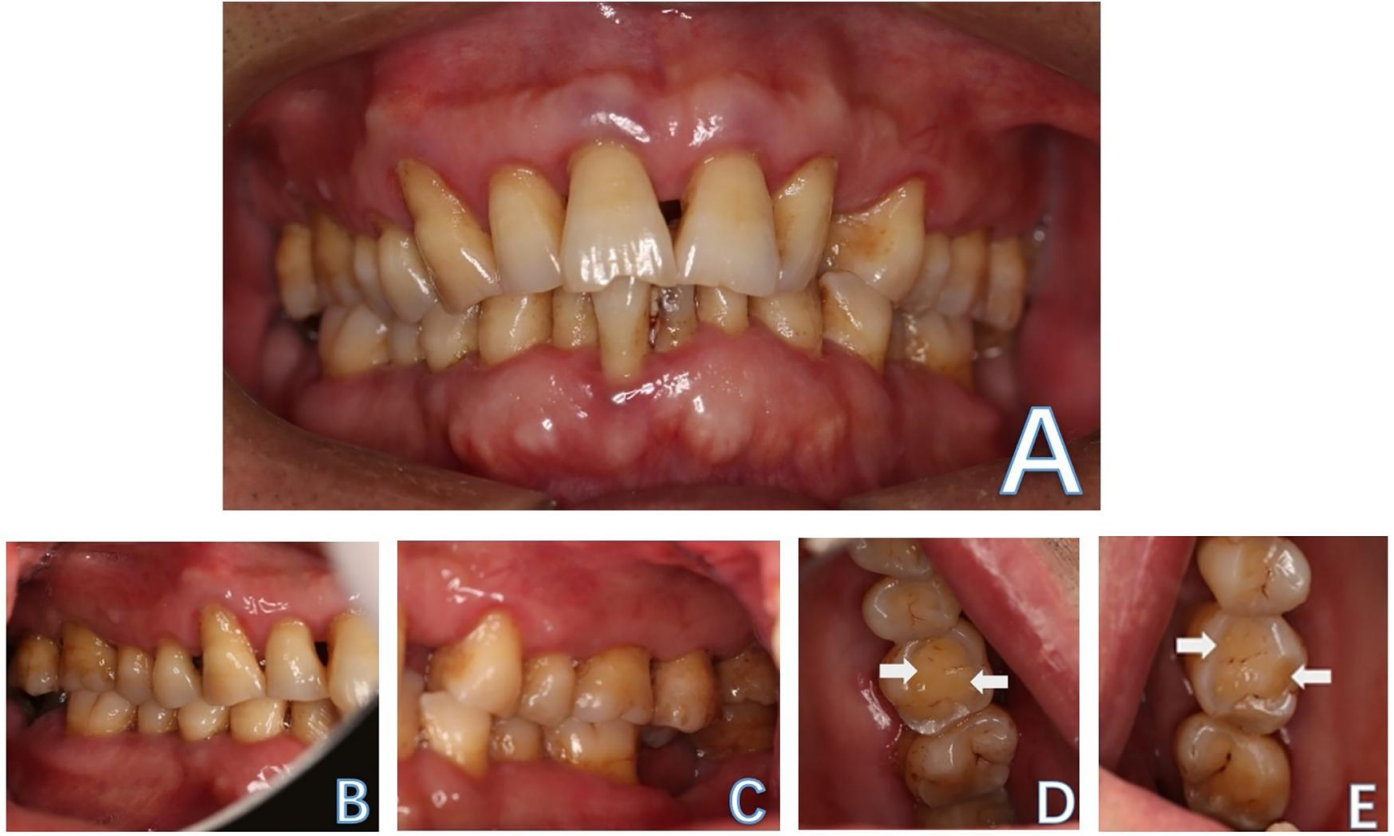
Intraoral examination of the oral cavity, frontal view **(A)**: right side **(B)**; left side view **(C)**; occlusal wear of the bilateral maxillary first molars **(D,E)** (arrow).

**Figure 2:**
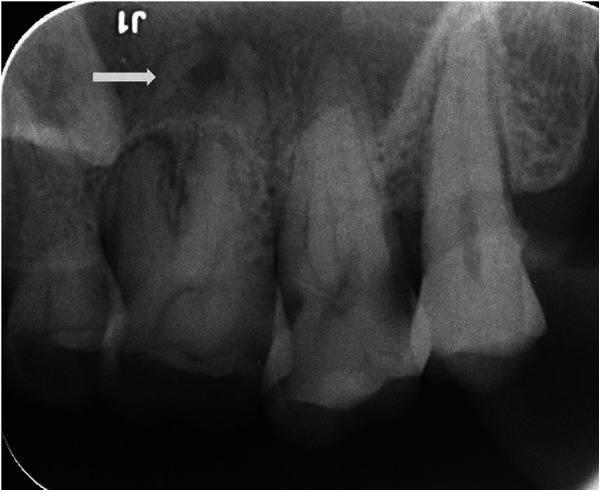
Periapical radiograph of the left maxillary first molar presented with a periapical radiolucency involving the apex at the palatal root and associated cemental tear.

**Figure 3:**
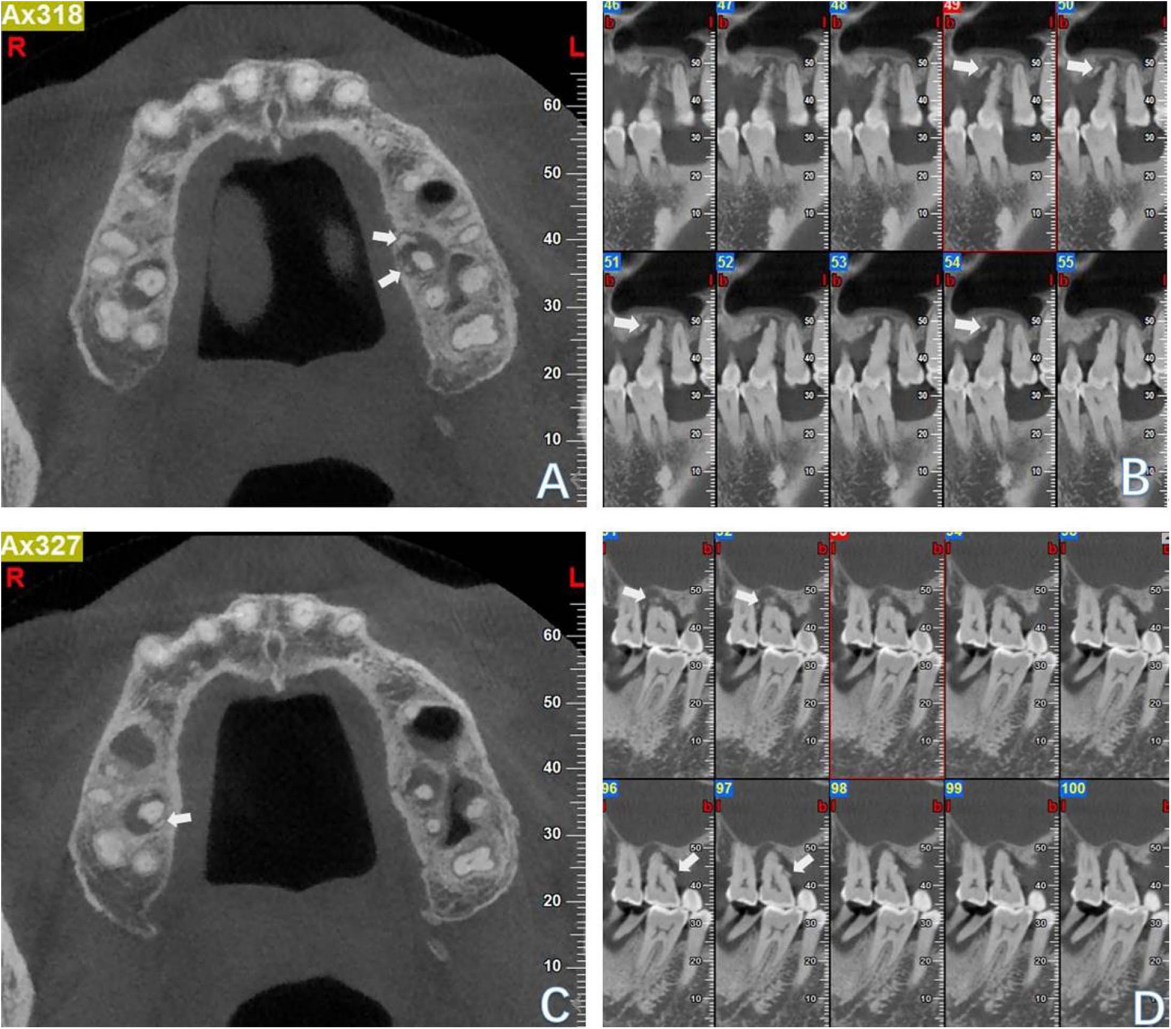
CBCT images of the left maxillary first molar **(A,B)** and the right maxillary first molar **(C,D)** in axial and sagittal views (arrows indicated the cemental tear).

A healthy 59-year-old man attended the Department of Cariology and Endodontology, Qingdao Stomatological Hospital affiliated to Qingdao University, China, with the chief complaint of gingival swelling and discomfort at his left upper quadrant while biting. The patient reported no history of drug allergy or any major systemic diseases. The dental history revealed that the patient did not receive regular dental check-ups and routine periodontal maintenance care. In addition, tooth #37 was extracted, and tooth #47 was fractured about three years ago; however, it was never restored (Figures 1A–C). The periodontal examination revealed the presence of generalized periodontal pockets (5–7 mm) with severe gum bleeding on probing. Periodontal pockets were also detected on the palatal aspect of tooth #26 and tooth #16 (8 mm and 7 mm deep, respectively). Teeth #26 and #16 with occlusal wear (Figures 1D,E) exhibit grade II mobility, while other teeth present with grade I mobility. No significant occlusal interference was detected during protrusive and lateral excursive movements. Since both of the patient's maxillary first molars exhibit cemental tears, the bilateral mandibular first molars were selected as control teeth. We used multiple methods for testing the pulp sensibility. The results of the maxillary first molars to thermal sensibility (heat and cold) test showed diminished responses compared to the control tooth. The results of the electric pulp testing: tooth #26:50, tooth #16:47, tooth #36:22 and tooth #46:24. The periapical radiograph showed a periapical radiolucency on the palatal root of tooth #26 (Figure 2). The CBCT of the maxillary first molars revealed cemental tears and associated excessive bone loss on their palatal aspects (Figures 3A–D). Based on these findings, it was diagnosed as cemental tear associated primary periodontal involvement. The differential diagnosis include endodontic periodontal lesion, root fracture, periapical cemental dysplasia or the laceration of alveolar bone.

**Figure 4:**
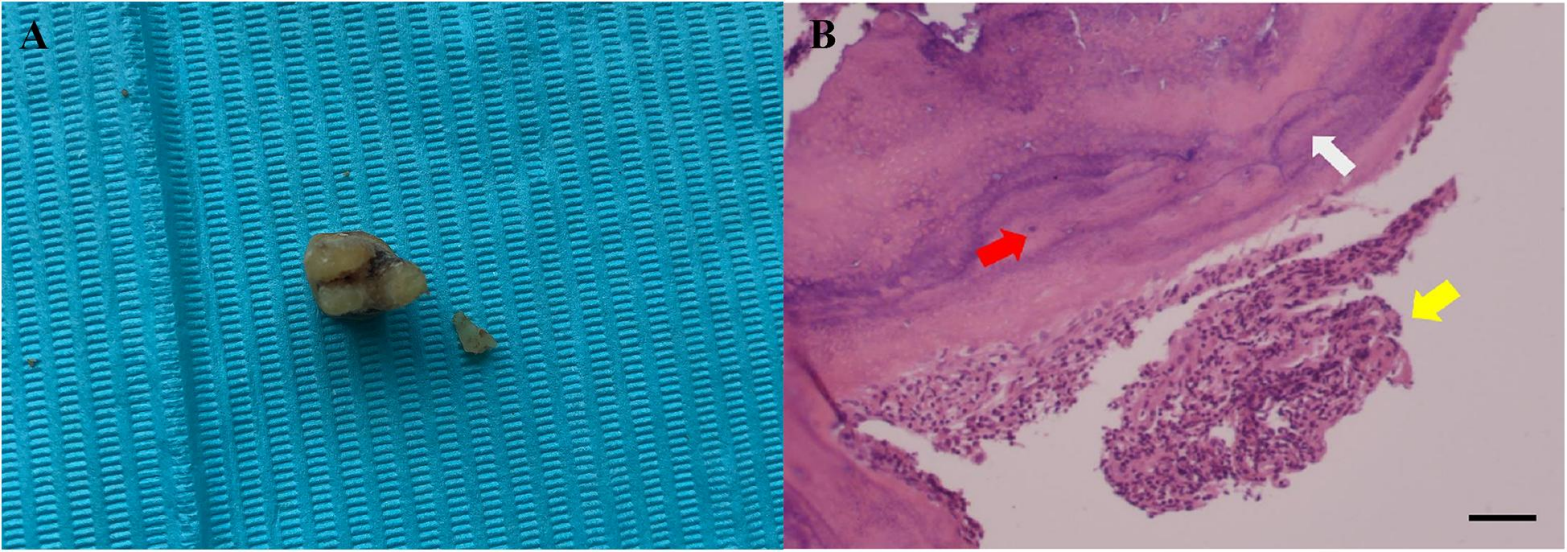
**(A)** The extracted left maxillary first molar and associated cemental fragment and **(B)** histological examination, the white arrow showing the cemental fragment, the red arrow showing cementocyte, and yellow arrow showing inﬂammatory inﬁltrate (hematoxylin and eosin stain × 100, scale bar: 100 *μ*m).

The initial treatment plan included supragingival and subgingival scaling, root planing, and flap surgery. Endodontic treatment was considered if the tooth's pulp vitality was lost during the treatment. However, the patient opted to extract tooth #26 and requested to postpone the treatment of tooth #16. In this case, the surgical procedure was performed by an oral and maxillofacial surgeon. After administering local anesthesia, tooth #26 and the fractured root fragments were extracted and sent for histopathologic evaluation (Figure 4A). The histopathological examination of the fractured root fragments from tooth #26 confirmed the diagnosis of a cemental tear (Figure 4B). At a two-month follow-up, the patient informed us that he had moved to another city. Tooth #16 had been extracted four weeks prior, and he was awaiting the placement of dental implants to replace the missing teeth.

## 3 Discussion

Cemental tear is a rare periodontal condition with an unknown etiology, posing a challenge for clinicians due to the delayed appearance of clinical signs (4). Moreover, it only becomes discernible on radiological examination once detached from the root surface. This study reported a case of bilateral cemental tears on the right and left maxillary first molars, leading to pain, discomfort, and periodontal destruction. The cemental tears were successfully managed with periodontal and surgical interventions. While cemental tears primarily affect single-root surfaces (approximately 75% of cases) (5), they may also affect the cervical, middle, and even apical third of the root surface of molars, as reported in this case.

Although the etiopathogenesis of cemental tears is not fully understood, various causative factors include occlusal trauma and periodontitis. Occlusal trauma includes acute or chronic trauma and primary or secondary trauma (6) resulting from excessive occlusal forces to the periodontium, which may lead to the formation of cemental tear (7). Primary occlusal trauma refers to damage caused by excessive or abnormal biting forces acting on healthy teeth or periodontal tissues, including nocturnal bruxism, premature tooth contact, severe occlusal wear, and so on (8). Secondary occlusal trauma occurs when normal or excessive forces are applied to teeth with insufficient periodontal support, particularly in patients with periodontitis. The loss of attachment leads to increased stress concentration, resulting in either increased tooth mobility or uneven distribution of stress (6). Furthermore, it results in a mismatch of applied forces at the cementodentinal junction and the cementum-periodontal ligament (9), which may exaggerate the localized bone resorption. In the present case, the patient reported a long history of biting hard foods, which led to the damage of the mandibular second molars a few years ago. Furthermore, the patient did not restore the damaged teeth, which might have concentrated the masticatory force on the first molars.

Periodontitis is not the direct cause of the cemental tear; however, it may cause the formation of cemental tear due to periodontal pockets or mechanical injury to the cementum by subgingival scaling (10). In this case, periodontal pockets (>5 mm) were detected in several teeth, including maxillary first molars. Long-term accumulation of bacteria in periodontal pockets results in cyclic demineralization and remineralization in the adjacent cementum, which remarkably reduces the strength of the affected cementum, thus promoting the occurrence of cemental tear (11).

The clinical or imaging findings of most cemental tears are easily confused with those of the following diseases, making accurate diagnosis clinically difficult, such as vertical root fracture (VRF), primary endodontic diseases, and primary periodontal diseases (6). The similarities between VRF and cemental tear include the presence of a narrow, deep periodontal pocket, percussion pain or pain on biting, and the possibility of occurring in either vital or non-vital teeth (12). However, during surgical exploration, we find that VRF is a complete split of the root, while a cemental tear is a superficial spalling. On radiographs, advanced vertical root fractures may show a clear, distinct fracture line running through the root. Additionally, VRF is more commonly seen in endodontically treated teeth with posts, while cemental tears occur more commonly in vital teeth (12).

Occlusal discomfort or pain is the common clinical feature of both cemental tear and primary endodontic diseases. But, the cemental tear can be distinguished from a primary endodontic lesion by pulp testing (13). In the early stage of a cemental tear, the pulp of the affected tooth is initially vital, however, if secondary infection occurs, the pulp subsequently loses its vitality. In contrast, teeth with primary endodontic diseases typically show abnormal pulp test results, most commonly being non-responsive, painful, or demonstrating a delayed response (14). In teeth with cemental tears, a narrow, deep, and isolated periodontal pocket that corresponds to the tear line can be detected. In contrast, periodontal probing in teeth with primary endodontic diseases is typically normal, unless it is a combined endodontic-periodontal lesion, in which case the pocket is usually wider (15).

Deep periodontal pockets, periodontal abscesses, and bleeding on probing are common clinical features shared by both cemental tears and primary periodontal diseases (5). In cases of cemental tear, symptoms such as probing depth and bleeding are strictly localized to a specific surface of a single tooth, where an isolated, narrow, and deep periodontal pocket extending to the apical region can be probed. In contrast, primary periodontal diseases typically affects multiple areas in the mouth, with varying severity, and the periodontal pockets are relatively broader (16).

The treatment of cemental tears varies depending on the extent of the lesion and the clinical presentation. Where possible, the torn fragment should be completely excised (8). Whereas, no active intervention is indicated if the cemental tear is merely a radiographical finding without any associated clinical signs and symptoms. The cemental tear formed at the coronal third of the root may be removed via subgingival scaling or root planing. A uniformly smooth surface promotes adhesion and regeneration of periodontal tissues (17). On the other hand, a surgical periodontal approach such as flap surgery is recommended to manage lesions present at the middle third of the root (18). The cemental tears affecting the apical third of the root require apical surgery and endodontic intervention (19). Lastly, extraction should be considered for teeth that are either not restorable or have a poor prognosis due to excessive bone loss.

Root canal treatment (RCT) is unnecessary for teeth without the involvement of the root apex. However, cemental tears in the apical third commonly lead to periapical lesions. The communication of these lesions with the oral cavity can subsequently cause pulp necrosis. If this occurs, root canal treatment and apical surgery will be necessary (8).

Guided tissue regeneration (GTR) is advocated in the treatment of cases with cemental tears (16, 20), because it involves the placement of a special bio-barrier membrane to selectively isolate and guide regenerative periodontal tissue cells to preferentially repopulate the root surface, thereby reconstructing the periodontal supporting tissues lost due to periodontal disease (14, 21). Other regenerative periodontal approaches have also been used, including the use of bio-regenerative factors such as enamel matrix derivatives (EMD) either alone or in combination with bone grafts and bio-barrier membrane (8, 22) The treatment plan for a cemental tear is typically formulated based on the location of the cemental tear in combination with the patient's preferences.

The choice of tooth extraction in the present cases was based on a comprehensive evaluation of several factors: severe bone destruction on the palatal root with grade II mobility and periodontal involvement. The cemental tear on the palatal root surface may provide insufficient support against occlusal forces. Most importantly, after communication with the patient, they actively chose extraction due to concerns regarding the financial cost, time commitment, fear, and uncertainty about the long-term outcome associated with conservative treatment options.

According to Lin and co-workers (12), maxillary and mandibular incisors were most frequently affected by the cemental tear. In the present case, rare bilateral maxillary first molars were involved and we confirmed this through histopathological examination. The limitations of the case report were that we were unable to conduct long-term follow-up and we should collect more such cases and use different treatments to evaluate their preventive and curative effects. Meanwhile, this case lacks an effective biological assessment of the patient's occlusal mechanics.

## 4 Conclusion

Within the limitation of this single case, we suggest that early diagnosis and comprehensive removal of the cemental tear are vital factors in treatment. Therefore, cemental tears should be considered in all teeth, including molars. Clinicians should remain vigilant when examining patients presenting with potential causative factors such as localized deep periodontal pockets, loss of attachment, or signs of occlusal trauma. Simultaneously, attention should be paid to distinguishing between cemental tears and root fractures.

## Data Availability

The raw data supporting the conclusions of this article will be made available by the authors, without undue reservation.
